# Piezo-surgery technique and intramuscular dexamethasone injection to reduce postoperative pain after impacted mandibular third molar surgery: a randomized clinical trial

**DOI:** 10.1186/s12903-021-01759-x

**Published:** 2021-08-11

**Authors:** Wissam Nehme, Youssef Fares, Linda Abou-Abbas

**Affiliations:** Neuroscience Research Center, Faculty of Medical Sciences, Lebanese University, Beirut, Lebanon

**Keywords:** Postoperative pain, Impacted mandibular third molar, Piezosurgery, Intramuscular Dexamethasone Injection

## Abstract

**Background:**

Surgical extraction of the impacted mandibular third molar is commonly associated with postoperative pain, swelling, and trismus. Usually, rotatory instruments like burs have been used for osteotomy, while Piezosurgery is an innovative technique introduced to overcome the weaknesses related to the conventional technique. In addition, Dexamethasone administration before the extraction of impacted third molars is an efficient way to reduce postoperative pain due to robust anti-inflammatory activity. The purpose of the study is to evaluate the effect of piezo-surgery and dexamethasone injection on postoperative sequelae after the surgical extraction of impacted mandibular third molars, and ultimately to compare their effect on reducing postoperative pain.

**Methods:**

A randomized controlled clinical trial was conducted with a sample of 80 patients. Participants were divided into four groups: Group 1 (Conventional rotatory), Group 2 (Conventional rotatory with 8 mg dose of dexamethasone 30 min before surgery), Group 3 (Piezo-surgery), and Group 4 (Piezo-surgery with 8 mg dose of dexamethasone 30 min before surgery). The outcome variables were surgical working time calculated in minutes, maximal mouth opening measured in millimeters using Vernier Caliper at baseline and day 3 and postoperative pain assessed using a Visual Analog Scale (VAS) on days 1, 3, and 7.

**Results:**

The surgical working time was longer in piezo-surgery groups compared with the conventional rotatory instruments groups (15.82 ± 3.47 vs 23.33 ± 2.54; *p* value < 0.0001). The lowest reduction in mouth opening between baseline and 3rd-day post-op was found in the Piezo-surgery with Dexamethasone group (mean difference = 5.0, SD = 3.9, *p* value < 0.0001) followed by the Piezosurgery without Dexamethasone group (mean difference = 5.8, SD = 4.5, *p* value < 0.0001) and the highest average was reported by the Conventional rotatory without Dexamethasone (mean difference = 9.7, SD = 4.5, *p* value < 0.0001. In the four groups, the mean pain score was highest on the 1st day and gradually decreased over the following days. Comparison of the 1st and 3rd postoperative pain between groups revealed a lowest mean pain score in the Piezo-surgery with Dexamethasone group, followed by Conventional rotatory with Dexamethasone group and a highest mean score in the Conventional rotatory without Dexamethasone group (*p* value < 0.0001).

**Conclusion:**

The association of Piezosurgery osteotomy and Dexamethasone intramuscular injection could be an effective combination to reduce postoperative pain and trismus after impacted third molar surgery.

*Trial registration*: NCT04889781 (https://clinicaltrials.gov/), Date of Registration: 17/05/2021 (retrospectively registered), https://clinicaltrials.gov/ct2/show/NCT04889781?term=NCT04889781&draw=2&rank=1

## Background

In early 1954, Mead defined an impacted tooth as a tooth that is disallowed from erupting into a normal position [1]. It is a tooth that is partially or entirely unerupted. Etiology may be multifactorial frequently due to adjacent teeth, thick covering bone or soft tissue, size of the maxilla or mandible with the subsequent deficiency of space in the mouth, the abnormal pathway of eruption, atypical locating of tooth bud, differential root evolution between the distal and mesial roots, or pathological lesions [2, 3]. Impacted teeth can cause food impaction, cavities, pericoronitis, pain, and the development of bone lesions [2–4].

Surgical extraction of the impacted third molar is one of the most performed surgical procedures in the field of Oral and Maxillofacial Surgery [5]. The incidence of third molars impaction ranges from 9.5 to 68% in young adults and these teeth frequently erupt between the ages of 17 and 21 years old [6–8]. Due to the amplified occurrence of lower third molars impaction, and the connection of many complications with these impacted teeth, assessment of third molars surgery in terms of indications, surgical techniques, symptoms, and postoperative sequelae becomes a necessity to offer patients high-quality care and minimize their suffering. The procedure difficulty may range from pretty easy to extremely difficult, depending on its depth, angulation, and the resistance of the surrounding bone [9]. Surgical removal of these teeth is typically correlated with postoperative pain, facial swelling, and trismus while complications such as infection, dry socket, inferior alveolar nerve, or lingual nerve injuries are less common to occur [10, 11].

One of the most perilous steps in mandibular third molar surgery is the removal of the covering bone or osteotomy, for which many techniques are used. The usage of hand tools such as osteotome, chisel, or gouge for bone procedures in oral surgery has a very long record. In daily practice, rotating instruments like drills are used for osteotomy during oral surgery. However, bone overheating and destruction to adjacent tissues are disadvantages correlated with the usage of these techniques, because they produce extremely high temperatures, which can launch peripheral osteonecrosis and impede bone regeneration and wound healing.

Several advanced methods have been introduced and tested in the extraction of the impacted wisdom teeth [12–14]. Marie and Jean Curie first presented piezoelectric equipment which uses ultrasound technology in 1880 [15]. Its application to oral surgery was first recommended in the late 1980s by Horton et al. However, a dedicated machine for this purpose was introduced recently by Vercellotti, an Italian surgeon to overcome the limitations of rotatory instruments in oral surgery. Also well-known as ‘pressure electrification’, it has been well-defined by the term ‘piezo’ derived from ‘piezein,’ meaning the pressure in the Greek language [16, 17]. The advantage of Piezosurgery is that it uses ultrasonic micro-vibrations to remove bone with minimal harm to the adjacent tissues, which leads to quick postoperative wound healing. Ultrasonic osteotomy might improve the efficiency of cuts and, directly, reduce the morbidity rate subsequent from iatrogenic injuries [18, 19].

In many recent studies, postoperative pain was compared in patients with impacted mandibular third molars treated by piezoelectric surgery or by rotary osteotomy technique; they concluded that the piezo-surgery osteotomy technique produced less facial swelling and less postoperative pain [11, 20, 21]. Thus, numerous authors have suggested using piezoelectric devices to achieve osteotomies as an alternative to rotary instruments [22, 23].

**Fig. 1:**
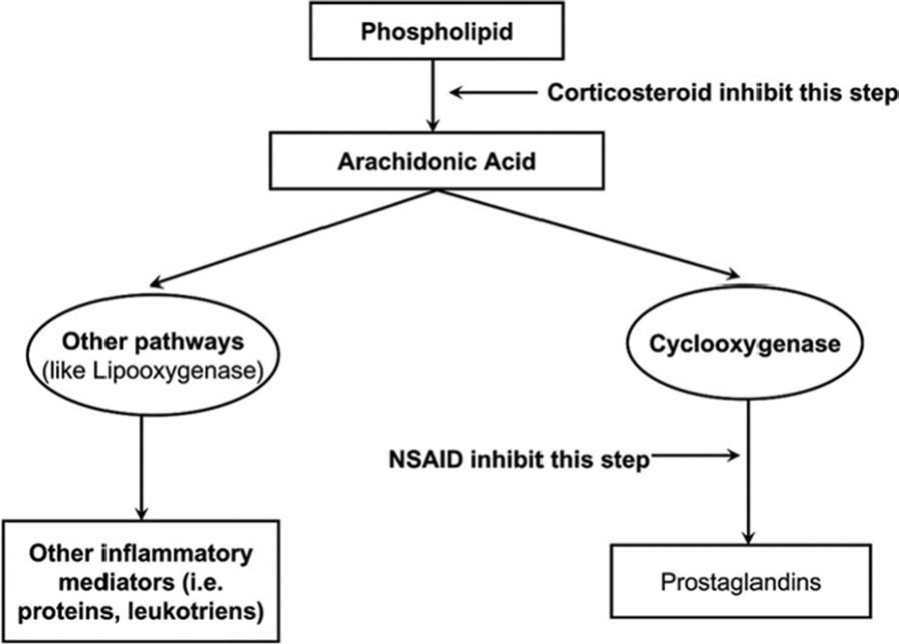
Mechanism of action of corticosteroids

Surgery-associated trauma initiates an inflammatory cascade, which activates biological reactions such as pain, swelling, and trismus [24]. A wide collection of drugs has been prescribed to prevent postoperative inflammation. Among these, corticosteroids are one of the utmost broadly used classes of drugs due to their solid anti-inflammatory action and relative safety in healthy patients [25–27]. Corticosteroids reduce inflammation by the repression of phospholipase A2, the primary enzyme involved in the transformation of phospholipids into arachidonic acid (Fig. 1) [26].

In addition, the use of anti-inflammatory medications is the prevalent attitude to decrease postoperative complications. Dexamethasone is known to be more powerful than other anti-inflammatory drugs because of its extended duration of action. It is related to an essential reduction of prostaglandins and leukotrienes; therefore, dexamethasone is one of the most frequently used corticosteroids [28]. Different routes of administration were used, such as oral administration, submucosal injection, and intramuscular injection, which have been shown to give equivalent results. However, the intramuscular route is popular in the field of oral and maxillofacial surgery due to its effectiveness and simplicity. Numerous studies have compared postoperative sequelae after the third molar in patients treated with or without dexamethasone injection; the majority confirmed that dexamethasone injection remarkably reduced postoperative pain, trismus, and facial swelling [25, 26, 29].

Several studies have evaluated the efficacy of the Piezosurgery technique and Dexamethasone injection separately [11, 20–23] but without evaluating their combined effect. Thus, the purpose of the present study is to evaluate the combined effect of piezo-surgery and dexamethasone injection on postoperative sequelae after the surgical extraction of impacted mandibular third molars, and ultimately to compare their combined effect on reducing postoperative pain.

## Methods

The study was approved by the Neuroscience Research committee at the Lebanese Faculty of medical Sciences. The clinical trial protocol was retrospectively registered in the Clinical Trial Registry of the U.S. National Library of Medicine (NCT04889781 https://clinicaltrials.gov/ct2/show/NCT04889781?term=NCT04889781&draw=2&rank=1. The results of this clinical trial are reported in accordance with CONSORT (Consolidated Standards of Reporting Trials) guidelines.

### Trial design and participants

A single-blind randomized controlled clinical trial was conducted over four months extending from February to June 2019. Individuals were selected to participate in the study which was conducted at the Central Military Hospital in Beirut. Inclusion criteria were healthy individuals with an orthodontic indication of bilateral upper and lower third molar removal, aged 15 to 30 years old, having mandibular impacted third molars, and a Pederson's Difficulty Score between five and eight. Exclusion criteria were as follows: Heavy smokers (≥ 20 cigarettes per Day); uncontrolled systemic conditions; acute infection of the surgical site; pregnant women; psychological problems; history of allergy to Dexamethasone, Amoxicillin, Mefenamic acid, or Acetaminophen.

### Interventions

Participants who were eligible to participate were randomly allocated to four groups of patients:

- Group 1 (Control): Surgical extraction using conventional rotatory instruments to perform osteotomy without Dexamethasone injection;
- Group 2: Surgical extraction using conventional rotatory tools to perform osteotomy with 8 mg intramuscular Dexamethasone injection 30 min before surgery;
- Group 3: Surgical extraction using the Piezosurgery technique without Dexamethasone injection;
- Group 4: Surgical extraction using the Piezosurgery technology with 8 mg intramuscular Dexamethasone injection 30 min before surgery.

### Study outcomes

The visual Analog (VAS), a measurement instrument that tries to measure a characteristic or attitude that is believed to range across a continuum of values, was used to subjectively evaluate the postoperative pain of the patient on days 1, 3, and 7. Scores are recorded by making a mark on a 10-cm line that represents a band between "no pain" and "worst pain" [5, 25].

The maximal mouth opening was measured at baseline and day three by measuring the space between the mesial incisal corners of the lower and upper right central incisors at the maximum mouth opening in millimetres (mm) using Vernier Caliper [13, 23]. The preoperative distance was considered as the baseline value.

### Sample size calculation

The calculation of the sample size was based on the primary outcome (postoperative pain) and the assumption of detecting a significant difference of 1 cm point in pain measurement on the VAS, an alpha of 0.05, and a power of 0.80. Based on the VAS mean results of a previous study conducted by Arakji et al. [30], the sample was determined to require at least 68 participants, 17 per group. Taking into account a probability of dropout or loss to follow up, a 10% was added to the sample size resulting in a minimal sample size of 76 male patients, 19 per group. Consequently, a sample size of 80 patients who required a surgical extraction of impacted mandibular third molar was recruited.

### Randomization

All patients were randomly assigned in four groups in a ratio of 1:1 using permuted block randomization via the website http://www.randomization.com. Participants were blind to the interventions assignment. Due to the difference between the four techniques, the allocation concealment of the researcher administering the interventions was not applied.

### Procedure

#### Preoperative phase

The medical history was checked for any previously unnoticed systemic problems. The difficulty score of the surgery was assessed according to Pederson's score on a panoramic X-Ray [31], and the maximum mouth opening (mm), was measured by the operator with a Vernier Caliper as the distance between the upper and lower incisors. All the patients were asked to do a Chlorhexidine 0.12% mouthwash 15 min before the operation [32, 33], and 40 patients (group two & four) received 2 ml of Dexamethasone Sodium phosphate 4 mg/1 ml (Dexamethasone Medis®) via IM route (Deltoid muscle site) half an hour before surgery.

#### Surgical phase

**Fig. 2:**
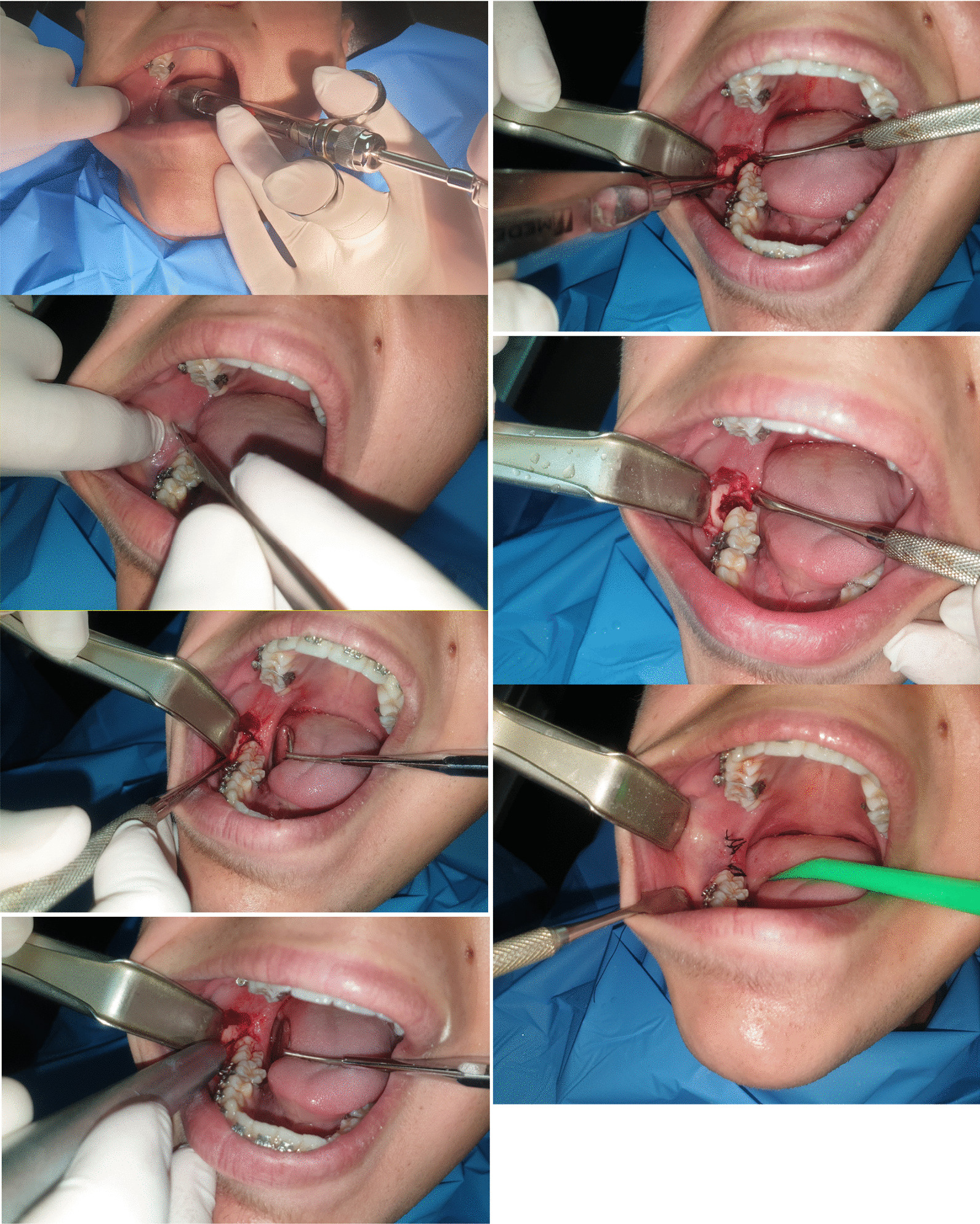
Step by step clinical images of the mandibular third molar extraction using carbide cylindrical drill

All extractions were performed by the same surgeon under local anesthesia consisting of 4% Articaine with 1:100,000 (UbistesinTM forte, 3 M ESPE, Germany); a full-thickness flap using a periosteal elevator (Molt Number 9) to uncover the impacted tooth and surrounding bone, and for group one and group two we use a new number 7 cylindrical carbide drill (DENTSPLY, USA) mounted on a surgical high-speed straight hand piece at 35,000 rpm accompanied by cooled saline solution irrigation; for group three and group four: Piezo-surgery device (PIEZOSURGERY® touch, MECTRON Medical Technology, Italy) was used for bone removal around the impacted tooth, using OT7 inserts. The frequency was adjusted between 25 and 35 kHz and the micro-vibration amplitude between 35 and 55 μm/s. In all four groups, natural non-absorbable 3/0 black silk sutures (BRAUN, Spain) were used to close the wound. The working time was calculated from the start of the incision until the end of the suturing and expressed in minutes (Fig. 2).

#### Postoperative phase

Ice application for 15 min/hour during the first 6 hours postoperatively was recommended [34, 35]. All patients received the same postoperative medication: 500 mg of Acetaminophen (Panadol by GSK) and 500 mg of Mefenamic acid (Ponstan*Forte by Pfizer), three times daily for three days [11, 28, 36]. For antibiotic prophylaxis oral Amoxicillin 500 mg (Ospamox® by Sandoz) was given, three times daily for five days [27, 37, 38] (No patients were allergic to penicillin in the study), and chlorhexidine gluconate mouthwash (Eludril Pro by Pierre Fabre), was recommended four times daily for seven days, the first day after the surgery, not the same day [32, 33].

### Statistical analysis

Data entry and analyses were performed using the statistical software SPSS version 22.0. Descriptive statistics were reported using means and standard deviations (SD) for continuous variables and frequency with percentages for categorical variables. Demographic Characteristics of the patients were compared across the four groups using the chi-squared test for categorical variables and ANOVA or Kruskal Wallis for continuous variables as appropriate. Bonferroni correction or Mann–Whitney U test on post hoc analysis was used for pairwise comparison. Paired T-test was used to compare the mean of mouth opening reduction between day 1 and day 3 for each group. Friedman test was used to compare the mean pain score across the four groups on days 1, 3, and 7. All statistical tests were two-sided, and the significance level was set at 0.05.

## Results

**Fig. 3:**
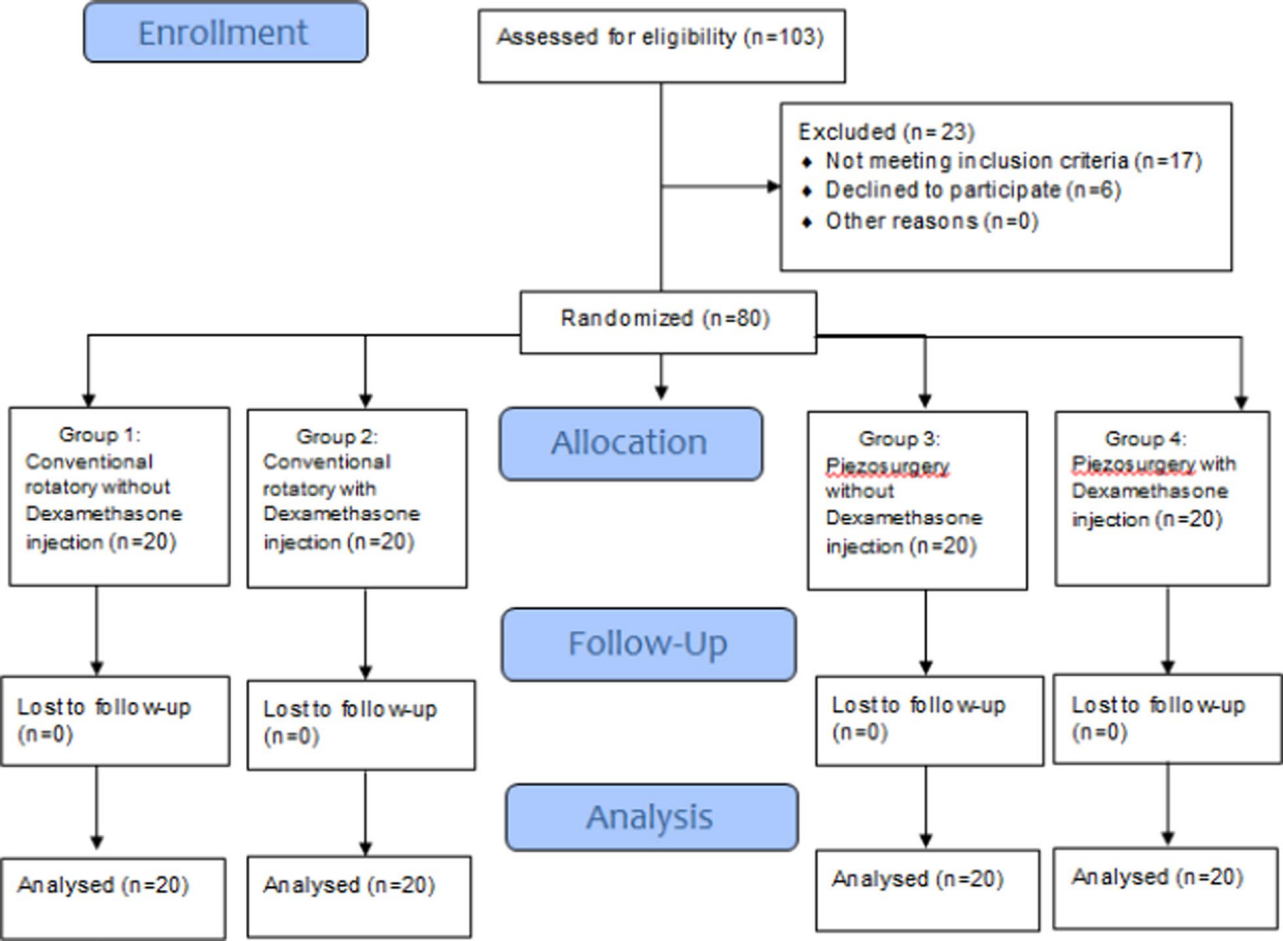
Flow diagram of participants

The flow of participants through the trial is shown in the CONSORT diagram (Fig. 3). A total of 103 patients were assessed for eligibility. Of these patients, 17 didn’t meet our eligibility criteria and 6 refused to participate. The remaining 80 patients were randomly assigned at a ratio of 1:1 by a simple randomization with no stratification. Consequently, 20 patients were assigned in each group. There was no loss to follow-up between groups. The total sample included 42 women (52.5%) and 38 men (47.5%). There were no significant differences among the four groups in terms of gender. Study patients' age range varied between 16 and 26 years, and the mean age was 19.9 ± 2.61. There were no statistical differences between the groups in terms of age distributions (P = 0.966). There were no statistical differences between the groups in terms of Pederson difficulty score (P = 0.951) (Table 1).

**Table 1:** Baseline characteristics and surgical working times of the study patients

	Group 1 n = 20	Group 2 n = 20	Group 3 n = 20	Group 4 n = 20	*p* value
Age (Mean ± SD)	19.8 ± 2.41	19.958 ± 2.35	19.88 ± 2.80	20.18 ± 3.00	0.966
Gender n (%)					0.801
Male	9 (45)	10 (50)	11 (55)	8 (40)	
Female	11 (55)	10 (50)	9 (45)	12 (60)	
Pederson difficulty score (Mean ± SD)	6.45 ± 1.09	6.35 ± 0.98	6.4 ± 1.09	6.55 ± 1.23	0.951

SD standard deviation, n frequency, % percentage, *p* value less than 0.05 is considered significant

Group1: Conventional rotatory without Dexamethasone

Group2: Conventional rotatory with Dexamethasone

Group3: Piezo-surgery without Dexamethasone

Group4: Piezo-surgery with Dexamethasone

Regarding the surgical working time, there was a significant difference between the four groups (*p* value < 0.0001) (Table 3). No significant difference was observed between group 1 and group 2 (*p* value = 1.00), and between group 3 and group 4 (*p*  = 1.00), since they shared the same surgical technique. The longest surgery time was seen in the piezo-surgery groups (*p* value < 0.05). Regarding mouth opening distance, there were no significant differences in mouth opening at baseline across the group. However, a statistical significance was found 3 days post-op across the four groups (*p* value = 0.016). Paired samples *t* test showed a reduction in mouth opening 3 days after the surgery in all four groups (*p* value < 0.0001). The lowest reduction in mouth opening between baseline and 3rd day post-op was found in the Piezo-surgery with Dexamethasone group (mean difference = 5.0, SD = 3.9, *p* value < 0.0001) followed by the Piezosurgery without Dexamethasone group (mean difference = 5.8, SD = 4.5, *p* value < 0.0001) and the highest average was reported by the Conventional rotatory without Dexamethasone (mean difference = 9.7, SD = 4.5, *p* value < 0.0001) (Table 2). Bonferroni test showed a significant difference in the reduction of mouth opening between group 1 and group 2 (*p* = 0.034) and between group 1 and group 3 (*p* = 0.047) as well as between group 1 and group 4 (*p* value = 0.011). However, there was no significant difference between group 2 and group 3 (*p* = 1.00) and between groups 2 and 4 (*p* value = 1.00) as well as between group 3 and group 4 (*p* value = 1).

**Table 2:** Comparison of surgical working time and mouth opening (mm) at baseline and day 3 between and within groups

	Group 1 n = 20	Group 2 n = 20	Group 3 n = 20	Group 4 n = 20	*p* value
Surgical Working time	15.9 ± 3.6	15.7 ± 3.5	23.2 ± 2.6	23.4 ± 2.5	< 0.0001
Mouth opening (mm)					
Preoperative	44.9 ± 6.5	45.4 ± 6.8	45.5 ± 5.9	45.1 ± 6.7	0.992
Post-op Day 3	35.0 ± 5.2	39.4 ± 6.3	39.8 ± 5.5	40.8 ± 4.2	0.016
Difference (Post-op Day 3 to preoperative)	9.7 ± 4.5	5.6 ± 4.3	5.8 ± 4.5	5.0 ± 3.9	0.005
*p*value	< 0.0001	< 0.0001	< 0.0001	< 0.0001	

Data are presented as mean and standard deviation, n frequency, *p* value less than 0.05 is considered significant

Group1: Conventional rotatory without Dexamethasone

Group2: Conventional rotatory with Dexamethasone

Group3: Piezo-surgery without Dexamethasone

Group4: Piezo-surgery with Dexamethasone

Among the four groups, the mean pain score was highest on the 1st day and gradually decreased over the following days (*p* value < 0.0001). Statistically significant reductions in pain levels from day 1 to day 3, and from day 3 to day 7 in all four groups (P < 0.0001) were found in all groups. Comparison of the 1st and 3rd postoperative pain between groups revealed a lowest mean pain score in the Piezo-surgery with Dexamethasone group, followed by Conventional rotatory with Dexamethasone group and a highest mean score in the Conventional rotatory without Dexamethasone group (*p* value < 0.0001). Concerning the 7th day, despite a similar trend, no statistical significance was revealed (*p* value = 0.064). The differences in pain scores at days 1, 3, and 7 among the groups were not found to be statistically significant (*p* value > 0.05) (Table 3).

**Table 3:** Pain levels between groups at 1st, 3rd, and 7th postoperative days between groups

	Group 1 n = 20	Group 2 n = 20	Group 3 n = 20	Group 4 n = 20	*p* value
Pain levels					
Post-op day 1	5.4 ± 1.2	4.15 ± 1.0	4.25 ± 1.0	3.3 ± 1.0	<0.0001
Post-op day 3	3.75 ± 1.5	2.8 ± 1.0	2.85 ± 1.1	2.2 ± 0.61	<0.0001
Post-op day 7	2 ± 2.07	1.1 ± 1.51	1.3 ± 1.55	0.7 ± 0.57	0.064
*p* value	< 0.0001	< 0.0001	< 0.0001	< 0.0001	
The difference in pain level					
Post-op day 3 to Post-op day 1	1.65 ± 1.27	1.35 ± 1.04	1.40 ± 1.14	1.15 ± 0.93	0.636
*p*value	< 0.0001	< 0.0001	< 0.0001	< 0.0001	
Post-op day 7 to Post-op day 1	3.40 ± 2.5	3.05 ± 1.82	2.95 ± 1.90	2.65 ± 1.03	0.703
*p*value	< 0.0001	< 0.0001	< 0.0001	< 0.0001	
Post-op day 7 to Post-op day 3	1.75 ± 2.5	1.70 ± 1.78	1.55 ± 1.32	1.5 ± 0.69	0.962
*p*value	< 0.0001	< 0.0001	< 0.0001	< 0.0001	

Data are presented as mean and standard deviation, n frequency, *p*value less than 0.05 is considered significant

Group1: Conventional rotatory without Dexamethasone

Group2: Conventional rotatory with Dexamethasone

Group3: Piezo-surgery without Dexamethasone

Group4: Piezo-surgery with Dexamethasone

The post hoc Bonferroni-test revealed a statistically significant mean difference between group 1 and group 2 (*p*value = 0.002) and between group 1 and group 3 (*p* value = 0.005) as well as between group 3 and 4 (*p* value 0.049) and between group 1 and group 4 (*p* value < 0.0001) in the 1st-day post-op. However, there was no significant difference between group 2 and group 3 (*p* value = 1.00), group 2, and 4 (*p* value 0.172) in the 1st-day post-op (Table 4). The 3rd day post-op revealed a statistically significant difference in the mean pain score between Conventional rotatory without Dexamethasone group and Conventional rotatory with Dexamethasone group (P = 0.028) and between Conventional rotatory without Dexamethasone group and Piezo-surgery without Dexamethasone group (P = 0.049), between Conventional rotatory without Dexamethasone group and Piezo-surgery with Dexamethasone group (*p* value <0.0001), and between Piezo-surgery without Dexamethasone group and Piezo-surgery with Dexamethasone group (*p* value 0.025). However, there was no significant difference between group Piezo-surgery without Dexamethasone group and Conventional rotatory with Dexamethasone group (*p* value = 0.95) as well as between Conventional rotatory with Dexamethasone group and Piezo-surgery with Dexamethasone group (*p* value 0.051). In the post-op 7 th day, no statistically significant differences in the pain score were found between the Conventional rotatory with Dexamethasone group and Piezo-surgery without Dexamethasone, and Piezo-surgery with Dexamethasone (*p* value > 0.05) (Table 4). No adverse events were observed or reported for any of the treatment groups.

**Table 4:** Mean pain score differences among the groups in the 1st, 3rd, and 7th postoperative days

	Day 1	Day 3	Day 7
Mean difference (95% CI) (Group1 vs. Group 2)	1.25 (0.34–2.16)	0.95 (0.01–1.89)	0.9 (− 0.411 to 2.21)
*p* value	0.002	0.028	0.043
Mean difference (95% CI) (Group1 vs. Group 3)	1.15 (0.24–2.06)	0.90 (− 0.036 to 1.83)	0.7 (− 0.61 to 2.01)
*p* value	0.005	0.049	0.16
Mean difference (95% CI) (Group1 vs. Group 4)	2.00 (1.09–2.91)	1.55 (0.61–2.48)	1.3 (− 0.01 to 2.61)
*p*value	< 0.0001	< 0.0001	0.04
Mean difference (95% CI) (Group2 vs. Group 3)	− 1.00 (− 1.02 to 0.81)	− 0.05 (− 0.98 to 0.86)	− 0.2 (− 1.51 to 1.11)
*p* value	1.0	0.95	0.50
Mean difference (95% CI) (Group2 vs. Group 4)	0.75 (− 0.16 to 1.66)	0.60 (− 0.33 to 1.53)	0.4 (− 0.912 to 1.711)
*p* value	0.017*	0.051	0.48
Mean difference (95% CI) (Group3 vs. Group 4)	− 0.85 (− 1.76 to 0.06)	0.65 (− 0.28 to 1.59)	0.6 (− 0.711 to 1.911)
*p* value	0.016	0.025	0.166

95%CI 95% Confidence interval, vs. versus, *p* value less than 0.05 is considered significant

Group1: Conventional rotatory without Dexamethasone

Group2: Conventional rotatory with Dexamethasone

Group3: Piezo-surgery without Dexamethasone

Group4: Piezo-surgery with Dexamethasone

## Discussion

Postoperative pain, facial swelling, and trismus are stressful conditions that are faced after impacted mandibular third molar surgery [28]. Thus, oral surgeons try to decrease postoperative complications via different approaches such as antibacterial mouthwashes [32, 33, 39], prophylactic antibiotics [40–43], new flap design [4], anti-anxiety medication [44], use of corticosteroids [25, 26, 29], and the use of piezosurgery. In addition, anti-inflammatory drugs are used to decrease postoperative complications. Dexamethasone is known to be more powerful than other anti-inflammatory drugs because of its extended duration of action [29, 45, 46]. It is related to an essential reduction of prostaglandins and leukotrienes; therefore, dexamethasone is one of the most frequently used corticosteroids [47, 48]. Different routes of administration were used, such as oral administration, submucosal injection, and intramuscular injection, which have been shown to give equivalent results [29]. However, the intramuscular route is popular in the field of oral and maxillofacial surgery due to its effectiveness and simplicity.

This clinical trial was designed to evaluate the effect of Piezosurgery and Dexamethasone on post-op outcomes after impacted mandibular third molar surgery and to assess their combined effect thoroughly. The surgical working time was measured from the start of the incision to the end of suturing. The time required to achieve the whole procedure was longer in piezo-surgery groups compared with the conventional rotatory instruments groups. This outcome is consistent with the results of the studies conducted by Barone et al. in 2010 in which the surgical working time was 30.5 ± 4.4 min in the rotatory group and 34.3 ± 7.4 min in the Piezosurgery group [49]. Another study conducted by Piersanti et al. in 2014 reported that the mean surgical time was 36.8 ± 10.6 min for piezosurgery and 30.8 ± 6.1 min in the conventional rotating hand piece. Same results were also reported by Arakji et al. in 2016 (28.5 ± 3.57 min for piezosurgery vs 17.6 ± 2.95 min for the rotary group with a significant *p* value of 0.0001) [11]. In addition, the differences between groups using the same technique were very far to reach a level of significance, which is expected because dexamethasone injection does not interfere with the surgical procedure. The trismus which was evaluated by assessing interincisal maximal opening at 3rd-day post-op showed a higher reduction in mouth opening in the first group (rotatory without Dexamethasone), and better results in the three other groups (Piezosurgery or/and Dexamethasone). Even if the Piezosurgery with Dexamethasone group showed the best effect on reducing trismus, but the difference remained statistically not significant with the second group (Rotatory with Dexa) and the third group (Piezosurgery without Dexa). Thus, using Piezosurgery or intramuscular dexamethasone injection or both have the same effect on the trismus three days after the surgery. Similar to our results, a clinical trial conducted by Goyal et al. in 2012 also showed a significant reduction of trismus in the Piezosurgery group on the 3rd, 5th, and seventh postoperative days [50]. Further, a clinical trial conducted by Piersanti et al., (2014) which assessed trismus on each day postoperatively showed better values for mouth opening on the 2nd postoperative day [21]. On the other hand, Sivolella et al. found that there was no significant difference in the maximal mouth opening between the Piezosurgery and rotary instruments on the 7th postoperative day [36]. Ehsan et al. reported a significant drop in trismus on the second postoperative day after dexamethasone injection, which is consistent with our study results.

Our findings revealed a highly significant reduction in postoperative pain on the 1st and 3rd day, and a statistically insignificant reduction on the 7th day, with Dexamethasone injection when compared to patients treated with the same technique without Dexamethasone. This decrease in pain is thought to be the outcome of the rise in the patient's pain answer initiated by the reduction of bradykinin level and the increase of endorphin level, resulting from steroidal actions. Also, there was a highly significant reduction in postoperative pain on the 1st and 3rd day, and a statistically insignificant decrease on the 7th day, with Piezosurgery osteotomy, compared to the rotating burr technique without or with Dexa injection. Postoperative pain is directly related to the manipulation of the tissues and the aggressiveness of the surgery, such as the sliding of the rotating bur and overheating. On the other hand, the reason which can explain the reduced post-op pain in the Piezosurgery groups is ultrasonic vibrations that permit selective and defined cuts, leading to an advanced level of accuracy and safety and less tissue harm than using traditional rotatory burs.

When we compare post-op outcomes between patients treated with Piezosurgery technique without Dexa injection and those treated with Dexamethasone injection with a conventional osteotomy, we found that post-op pain scores were relatively higher in operations using Piezosurgery without Dexamethasone injection, but the difference was not significant. Finally, group four where patients were treated using both Piezosurgery and Dexa injection showed better post-op outcomes when compared to the three other groups at 1st, 3rd, and 7th post-op. Our results were consistent with many recent studies. In 2016, Arakji et al. compared the effects of Piezosurgery and conventional rotary instruments for removal of impacted mandibular third molars, and concluded that piezosurgery reduces postoperative pain, trismus, and swelling and enhances the postsurgical quality of patient’s life [11]. In 2019, Gumrukcu et al. also performed a retrospective clinical study to compare the post-operative effects of the conventional surgery, piezo surgery technique and submucosal dexamethasone injection in lower third molars extractions, and concluded that dexamethasone was effective in reducing swelling, pain, mouth opening restriction, and that Piezosurgery has positive effects on pain, swelling, trismus, and number of used analgesics compared to the control group [28]. In 2021, Al-Delayme et al. compared piezo-electric surgery with conventional and concluded that piezosurgery reduces postoperative pain, trismus, and swelling [51]. Furthermore, Gulnahar et al. compared postoperative morbidity between piezoelectric surgery and conventional rotary instruments in mandibular third molar surgery, and concluded that piezosurgery is a safe alternative method that can be used for the removal of impacted mandibular third molars, but did not provide a significant benefit in terms of postoperative pain and trismus [52].

Strengths of the present study include the high-quality study design (RCT), the randomization, the similarities of the baseline characteristics across the four groups, the blindness of the patients, the use of a well-validated instrument, and procedures to collect information about the outcomes of interest, the substantially larger sample size compared to previous studies, and the application of the techniques by the same operator. Limitations of the study were that double-blinding was not possible, the operator was not masked to allocation but this is an unavoidable limitation. Another weak point is the lack of data collected concerning the need for dental separation or not, the hormonal influence on girls, facial swelling, and analgesics intake, bearing in mind that all of these factors can have an impact on postoperative pain and interfere with the final results, especially if painkillers are taken more or less than prescribed. Therefore, we recommend designing a study taking into consideration all these facts.

## Conclusion

Regardless of increasing the surgical working time than conventional rotatory technique, Piezosurgery significantly reduces the related postoperative sequelae of third molar surgery and hence improves the quality of life of the patient. Piezosurgery is also recommended when the third molar has particularly dangerous or unusual positions. As well, intramuscular Dexamethasone injection half an hour before surgery reduces postoperative pain and trismus. Overall, the comparable results showed that Intramuscular Dexamethasone injection and the use of the Piezosurgery device might have the same effect on reducing post-op pain in impacted mandibular third molar surgery. The association of Piezosurgery osteotomy and Dexamethasone intramuscular injection could be an effective combination to reduce postoperative pain and trismus after wisdom tooth surgery. More studies of large sample sizes are necessary to validate these findings.

## Data Availability

The datasets used and analysed during the current study are available from the corresponding author on reasonable request.

## Abbreviations

C.N.S.: Central Nervous System
Dexa: Dexamethasone
g.: Gram
Gr.: Group
I.M.: Intramuscular
M.O.: Mouth Opening
min: Minutes
mm: Millimetre
P: *p* value
PoSSe: Subjective Scale of Severity of Postoperative Symptoms
Post-op: Postoperative
Rpm: Round per Minute
